# Editorial: Physisorption of Gases: Adsorbent Characterization, Adsorbent-Adsorbate Equilibrium and Kinetics

**DOI:** 10.3389/fchem.2021.668553

**Published:** 2021-04-06

**Authors:** Alexander M. Puziy, Peter Lodewyckx, James A. Ritter, Armin D. Ebner, Fabian Suarez-Garcia

**Affiliations:** ^1^Institute for Sorption and Problems of Endoecology, National Academy of Sciences (NAS) of Ukraine, Kyiv, Ukraine; ^2^Royal Military Academy, Brussels, Belgium; ^3^Department of Chemical Engineering, University of South Carolina, Columbia, SC, United States; ^4^Instituto de Ciencia y Tecnología del Carbono INCAR-Consejo Superior de Investigaciones Científicas (CSIC), Oviedo, Spain

**Keywords:** adsorption, characterization, porous structure, kinetics of adsorption, dynamics of adsorption

The purpose of this Research Topic is to get an overview of new advances in the synthesis of new selective adsorbents that can reduce greenhouse gas emissions; in methods of surface modification of adsorbents and their influence on adsorption properties; and also to show new methods of characterization of porous structure and surface properties in terms of the pore size distribution and adsorption energy distribution calculated from experimental data on gas adsorption.

To reduce the emissions of greenhouse gases, particularly of CO_2_, ionic liquid/metal-organic framework composites, using ZIF-8 as the original material, were synthesized by Ferreira et al.. For the entire range pressure investigated, i.e., 0.5 and 16 atm, the prepared composites showed superior volume capacities and CO_2_ selectivities over N_2_ or CH_4_ relative to pristine ZIF-8. In particular, the composite [C_4_MIM]_2_[MnCl_4_]@ZIF-8 exhibited a different low-pressure selectivity trend from the original ZIF-8, with a 33% increase in the CO_2_/N_2_ selectivity at 1 bar and a 19% increase in the CO_2_/CH_4_ selectivity at 10 bar. This material shows potential for use in a post-combustion CO_2_ capture application that can contribute to greenhouse gas mitigation.

The impact of fluorination on the texture and surface chemistry of activated carbon fiber (ACF) was studied by Velasco et al.. It has been shown that mild fluorination not only can preserve most of the textural properties of the parent ACF but also enhance the water uptake at the first stages of the water sorption process, together with a shift in the upswing of the water isotherms toward lower relative humidity. On the contrary, a higher concentration of fluorine has led to highly fluorinated fibers with lower porosity and a more hydrophobic character. Moreover, they presented lower chemical stability as demonstrated by a change in the shape of the water isotherms after two consecutive measurements. The kinetics of water sorption in the ACFs provided further insights into the different sorption phenomena involved. Hence, water sorption can help to tailor the water affinity, stability, and performance of fluorinated porous carbon materials under humid conditions.

An efficient method to characterize pore size distribution (PSD) of activated carbons was proposed by de Oliveira et al.. The method chooses a limited number of representative pores, which will constitute a simplified kernel to describe the PSD which is later applied to predict the adsorption equilibrium of other gases. The method allows quick solutions for large-scale calculations for carbonaceous materials screening. It also gives access to an easily understood and prompt evaluation of the structure-property relationship of activated carbons. The authors applied this method to the removal of organic contaminants in dilute aqueous solutions.

A theoretical and mathematical methodology based on the condensation approximation approach is described by Burhan et al.. The method intends to analyze the structure of various adsorbents in terms of the distribution of their adsorption energy sites. The use of this method for the analysis of nitrogen adsorption isotherms on various adsorbents was demonstrated.

The Guest Editors of this Research Topic are grateful to all the authors for the submitted articles and to all the reviewers for their comments on the manuscripts. We hope that readers will find the articles included in this Research Topic interesting and useful for their research. Finally, we are grateful to the editorial staff of Frontiers in Chemistry for their work in publishing this issue of Research.

## Author Contributions

All authors contributed to manuscript revision, read, and approved the submitted version.

## Conflict of Interest

The authors declare that the research was conducted in the absence of any commercial or financial relationships that could be construed as a potential conflict of interest.

